# Newspaper reporting of homicide-suicide and mental illness

**DOI:** 10.1192/pb.bp.114.049676

**Published:** 2015-12

**Authors:** Sandra Flynn, Linda Gask, Jenny Shaw

**Affiliations:** 1 University of Manchester, UK

## Abstract

**Aims and method**

To explore the portrayal of homicide-suicide in newspaper articles, particularly how mental illness was reported. We carried out a qualitative study in England and Wales (2006-2008). Data from newspaper articles obtained via the LexisNexis database were used to examine a consecutive series of 60 cases.

**Results**

A fascination with extreme violence, vulnerable victims and having someone to blame made homicide-suicides newsworthy. Some offenders were portrayed in a stereotypical manner and pejorative language was used to describe mental illness. The findings showed evidence of inaccurate and speculative reference to mental disorder in newspaper reports.

**Clinical implications**

The media should avoid speculation on people's mental state. Accurate reporting is essential to reduce stigma of mental illness, which may in turn encourage people to seek help if they experience similar emotional distress.

Homicide-suicide refers to an individual who commits homicide and subsequently takes their own life. There is no standardised definition regarding the time between the two incidents. A 3-day period has previously been taken as a cut-off point, the rationale being that a 24-hour cut-off is too restrictive. Mental state is likely to remain the same over a period of days and the two events are therefore connected, but reasons for the suicide may begin to change in excess of 3 days.^1,2^ These incidents attract widespread coverage in local and national newspapers. Consequently, the way they are reported informs both the general public's understanding of these incidents and their attitude towards offender and victim.^3-5^ Therefore, it is important that the circumstances are reported accurately, particularly when incidents involve people with mental illness. For instance, there is an association between certain methods of reporting suicide and increased suicide risk in young people and vulnerable adults (i.e. contagion).^6-8^ In addition, it has been uncovered that 14% of articles in the print media in 2011 referred to people with mental illness as a ‘danger to others’,^9^ reinforcing the stereotype that people with mental illness are violent. Speculating on a person's mental state without evidence is as stigmatising as the use of derogatory language. The UK ‘Time to Change’ campaign, led by the charities Mind and Rethink Mental Illness, aims to encourage the media to promote positive attitudes towards mental illness.^10^ If successful, this may encourage people to seek help.

This is the first study to examine newspaper reporting of mental illness in homicide-suicide. We aimed to explore how UK newspapers reported incidents of homicide-suicide and how mental illness was reported in relation to these cases.

## Method

### Study design

The findings presented are from a larger, mixed-method study of homicide-suicide undertaken by the National Confidential Inquiry into Suicide and Homicide by People with Mental Illness (NCISH) (details available from the authors on request). In brief, 60 cases of homicide-suicide were reported to NCISH by the Home Office Statistics Unit of Home Office Science and by individual police services. The offences occurred between 1 January 2006 and 31 December 2008 in England and Wales. The inclusion criteria for these incidents were that the suicide occurred within 3 days of the homicide and that a coroner returned a verdict of unlawful killing for the victim and suicide/open verdict for the offender. Additional information on each case was sought from coroner's files, police records, newspapers, general practitioner medical records and mental health service records. A diagnosis of mental disorder was determined from the offender's medical records.

### Data collection and sample

A search was undertaken for newspaper articles pertaining to the 60 cases of homicide-suicide. The LexisNexis database (www.lexisnexis.com) was consulted to locate articles published between January 2006 (the date of the first offence) and September 2012 (the date when analysis commenced), allowing time for the legal process to be concluded and reported in the media.

Searches of all local and national UK newspapers were undertaken, including tabloid and broadsheet publications, which ensured the data were representative and encapsulated a range of journalistic styles and biases. The victims' and offenders' names were used as search terms rather than the generic phrase ‘homicide-suicide’. In instances where individual searches yielded thousands of returns owing to common names, the term ‘suicide’ or ‘homicide’ was added to the search. In instances where there were no returns or the count was low, alternative spellings, name variants or known aliases were used. In six incidents where no articles were returned on LexisNexis, an additional search of online news media was undertaken.

### Missing data

After an extensive search, newspaper reports were obtained on 54 incidents (90%); no articles were found on 6 incidents (10%). It is possible that these events were not reported in the media; a previous study found only 62% of homicide-suicides were reported in national newspapers.^11^ Consequently, we felt that 10% missing data was within an acceptable range that would not bias the findings and would retain the generalisability of the results.

### Quantitative analysis

Descriptive statistics were generated to provide context for these homicide-suicide incidents and limited information has been presented. The results were reported using 95% confidence intervals. The analysis was undertaken using Stata version 12. If an item of information was not known for a case, the case was removed from the analysis of that item; the denominator in all estimates was the number of valid cases for each item and it indicates the number of missing cases per item.

### Qualitative analysis

The analysis of documents was undertaken in accordance with the principles set out by Hodder (2003).^12^ A systematic and comprehensive approach was adopted by applying framework analysis which involves five key stages: familiarisation, identifying a thematic framework, indexing, charting, and mapping and interpretation.^13^ Each article was read and an iterative coding process was undertaken until saturation was reached. The themes were subsequently refined into three domains. The coding was carried out by S.F. and themes were discussed with L.G. and J.S.. Data were analysed using MAXQDA version 10 (www.maxqda.com). The approach differs from other qualitative analysis methods in stages four and five as it enables the data to be managed into a series of matrices whereby the data can be explored by theme and by case.^14^ This systematic, yet flexible, approach was preferred to other methods owing to the volume of data collected. Alternative methods to analyse media coverage used in previous research, such as content analysis with predetermined ‘deductive’ coding structures,^15^ latent class analysis^16^ or multivariate analysis,^17^ were not considered appropriate for this data.

### Confidentiality and anonymity

The main data source were newspaper articles. Details of these incidents are therefore in the public domain.

### Ethical approval

The study received the Medical Research and Ethics Committee (MREC) approval on 9 April 2008 and is registered under the Data Protection Act. The study was granted exemption under section 251 of the National Health Service Act 2006 (formerly Section 60 of the Health and Social Care Act 2001), enabling access to confidential and identifiable information without informed consent in the interest of improving patient care (approved 23 October 2008).

## Results

### Description of the sample

Over a 3-year period (2006-2008), 60 incidents of homicide-suicide were identified in England and Wales. Newspaper articles were obtained on 54 (90%) of these cases. Overall, 16 323 articles were found on these incidents. Duplicate articles (repeated in later editions) were excluded and the content was filtered for relevance. A total of 1163 articles were used in the analysis, an average of 22 per incident (range 0-115). The characteristics of offenders and victims are presented in Table 1. Most offenders were male, with a median age of 44, and over a quarter were from a Black and minority ethnic group. The victims were most commonly the offenders' spouse/partner or ex-spouse/partner, or their child. Nearly two-thirds of the offenders had a history of mental disorder.

**Table 1:** Characteristics of offenders and victims

	*n* (%)	95% CI
Offender (*n* = 60)		
Median age, years (range): 44 (18–85)			
Gender: male	53 (88%)	80–97
Black and minority ethnic group	17 (29%)	17–41
History of mental illness from medical records	33 (62%)	49–76

Victim (*n* = 70)		
Median age, years (range): 38 (1–85)		
Gender: female	54 (77%)	67–87
Black and minority ethnic group	14(18%)	10–30
Relationship to the offender:		
Spouse/partner or ex-spouse/partner	45 (64%	53–76
Child	20 (29%)	18–39

Three themes emerged from the qualitative analysis: characteristics that made these incidents newsworthy: how homicide-suicides were reported and the accuracy of newspaper reports of mental illness compared with the information contained in the deceased's medical records (Table 2).

**Table 2:** Themes emerging from newspaper analysis

	Sub-theme
Theme 1: What makes homicide-suicide newsworthy?	Fascination with extreme violence and personal tragedy Characteristics of victims and offenders Having someone to blame
Theme 2: How are homicide-suicides reported?	Offender stereotypes The offender's personality Mental illness
Theme 3: Accuracy of newspaper reports of mental illness	Speculation that the incident was motivated by mental illness

### What makes homicide-suicide newsworthy?

#### Fascination with extreme violence and personal tragedy

Tabloid newspapers exhibited a fascination with the level of violence involved in the incident. More often this referred to the violence against the victim in the homicide rather than the suicide. Graphic descriptions were used in the headlines, presumably in an attempt to attract readers. An example of a headline that illustrates the sensationalist nature of the reporting is: ‘CRAZED; EXCLUSIVE: Dad hacks toddler son to death and then kills himself’ (*The Mirror*, 2 September 2006).

#### Characteristics of victims

The newsworthiness of these incidents was also associated with the characteristics of the victim. The deaths of vulnerable or innocent victims added a further tragic element to the story. In addition to labelling the victims and offenders as good or evil, the status of the victim was elevated due to their profession. When reporting on the deaths of two police officers, one in the line of duty while responding to a serious incident, the language used emphasised the bravery of the victim while simultaneously showing disdain for the offender:

HERO cop [victim] was shot dead yesterday when a gunman went berserk during a furious row with his girlfriend. [The victim] was part of a police armed response unit called out to a domestic dispute after crazed [offender] armed himself with a hunting rifle (*The Sun*, 4 October 2007).

#### Apportioning blame

There was increased newspaper coverage when the details of the homicide-suicide were used to highlight failure by services. In one article, the offender's personal responsibility was marginalised and the focus turned towards perceived institutional failings, for which the newspaper blamed the prime minister:

‘Perhaps our PM and members of his government might like to imagine some inept social services bod bursting into THEIR home uninvited and removing their partner by force, saying: “It'll be better for everyone.” What's better for old people is that they feel safe and secure, and how the hell can they feel that when social-services Nazis tear them away from the one person left in the world who loves and understands them? The only person who remembers them as they were – strong and vibrant – not dependent on a state that doesn't give a stuff about them?’ (*News of the World*, 18 May 2008)

### How are the homicide-suicides reported?

We found markedly different styles of reporting between broadsheet and tabloid newspapers. A considerable number of articles reported short, factual accounts of the incident. By contrast, where the reports were opinion-based, these articles provided valuable insight as to how the offenders were perceived and portrayed to the general public.

#### Offender stereotypes

The portrayal of homicide-suicide in the media seemed to reinforce stereotypes and oversimplify the context of these events. For example, it was common for elderly couples with declining health to be described as being ‘devoted to each other’. Journalists assumed an empathic attitude toward the offender and the couple's situation in general. Commonly referred to as ‘mercy killings’, a similar sympathetic tone was observed in cases of filicide by mothers where a child was killed for perceived altruistic reasons. However, fathers who killed their children did not receive the same level of sympathy, even when they experienced similar emotional distress before the homicide. In one article, the newspaper reported a mother's defence of her son's actions, in which two of her grandchildren died. This sentiment was subsequently criticised in the article, presumably to reinforce the message to the reader that there was no excuse for the offender's actions and he did not deserve any sympathy.

#### The offender's personality

Direct quotations from family and friends were commonly used to generate a profile of the offender. These descriptions provided insight into how the person was perceived, and consequently, the image created of the offender in the media. Each case of homicide-suicide generated numerous articles in a range of publications. The witness descriptions of the perpetrator differed depending on the newspaper and the informant quoted. The contrast in the portrayal of the same offender is demonstrated:

‘Everyone is stunned and no one can believe it. He was such a nice bloke, he'd do anything for anyone and was very helpful and he absolutely loved his children.’ (*Daily Telegraph*, 23 September 2008)

‘There was something weird about him. I knew [he] wasn't right in the head. He was an attention-seeking control freak who had a thing about teenage girls.’ (*News of the World*, 28 September 2008)

#### Mental illness

Comments regarding the offender's perceived mental state were prominent in several newspaper headlines. Whereas most descriptions were written with sensitivity, there were some exceptions to this, notably from the tabloid press: ‘Nut free to kill for 3rd time’ (*The Sun*, 18 March 2006), ‘PSYCHO DADDY; Father strangled mum of his 4 kids then hung himself at home’ (*The Mirror*, 12 March 2009).

### Accuracy of newspaper reports of mental illness

#### Speculation that the incident was motivated by mental illness

Newspapers are produced for commercial reasons and articles are written for specific audiences. We found the majority of the homicide-suicide incidents involving people with a history of mental illness were reported responsibly and newspapers did not stigmatise the offender. However, it was observed that some newspapers published speculative comments concerning the offender's mental state, without being able to substantiate these claims:

‘She must have had a very troubled mind to do what she did. We can't imagine why she said to people she had cancer; she may have been suffering from some sort of mental illness. We are not aware of any mental health issues but that is something we shall be looking into.’ (*Birmingham Evening Mail*, 12 December 2007)

‘I would describe him as a psychopath. I saw him attack his brother with a hammer then run after him with a knife in the street.’ (*Yorkshire Post*, 9 March 2009)

In addition to the speculation regarding diagnoses, reporters often seemed to select quotations from witnesses that provided a default assumption of mental illness when there was seemingly no other plausible explanation. For example, they referred to the offender having ‘cracked’, ‘snapped’, ‘flipped’ or ‘gone berserk’. Although these terms appear in direct quotations from witnesses who knew the offender, the words imply the offender had experienced a mental health crisis at the time of the offence, yet no supporting evidence was provided to substantiate this.

## Discussion

### Newspaper interest in homicide-suicide

We found that homicide-suicides were highly newsworthy, with 90% reported in national and local newspapers, an average of 22 articles per incident. There are aspects of these offences which made them of public interest, notably they involved multiple victims, the majority of whom were intimate family members, consistent with previous research.^18^ Our data showed that extreme violence towards the victim, characteristics of the victim and the perceived failure of services to intervene in certain circumstances added to their media appeal. Emotive language, particularly in headlines, was used to attract the attention of the reader, which was consistent with findings from previous studies.^17,19^

### Reporting mental illness responsibly

In this study, we found the complexity of the events was often lost in the reporting. People who committed these acts were often assigned labels and portrayed in a stereotypical manner. We found evidence of derogatory language used to describe mental illness, such as ‘nut’ or ‘psycho’, although the majority of articles referred to people with mental illness more sensitively. This finding is consistent with a recent study undertaken by researchers at the Institute of Psychiatry in the UK which showed a decrease in the number of articles using pejorative language and referring to people with mental illness as being dangerous. The data also showed a simultaneous increase in anti-stigmatising newspaper articles and positive mental health promotion. However, the research reported no overall change in the proportion of stigmatising articles between 2008 and 2011.^15^

### Speculating on offender's mental state

Evidence of newspaper speculation on an individual's mental state without any corroborating medical evidence was an important finding of this study. Labelling offenders as ‘psychopaths’ not only stigmatises the deceased, it also causes distress to the surviving family members. Previous research has shown how relatives of people who carried out a homicide or suicide experienced additional anguish due to the person's portrayal in the media.^20,21^ Even where the evidence is lacking, reporters chose to reinforce the perception that mental illness is the only credible explanation for the offender's actions. This conjecture promotes a widespread belief that ‘all’ people who commit homicide-suicide must have been mentally ill, when in many cases mental illness was not a feature. Data from the larger study of homicide-suicide have shown that 38% of offenders had no history of mental illness, consistent with a similar study in the USA.^22^ Guidance published by the American Foundation for Suicide Prevention and partners suggests that careful newspaper coverage could help to change these misconceptions.^23^ Similarly, guidance for the media by Time to Change^10^ suggests to journalists: ‘Don't speculate about someone's mental health being a factor in the story unless you know it to be 100% true’. A further ‘reporting tip’ asks journalist to consider: ‘Who are your sources? Can you rely on eyewitnesses or neighbours to provide facts or has an assumption been made about someone's mental health status?’ Examples of language that should or should not be used to avoid the perception of dangerousness are also detailed.

### Limitations

Newspaper databases such as LexisNexis have been criticised for not being comprehensive and have been described as inconsistent and incomplete.^24^ Previous research has shown that content (i.e. news wire stories) could have been removed before archiving; consequently, the original news content could be different from the archived version, which can introduce error.^24^ Restrictive search terms could also lead to articles being missed. However, in this study the use of the individuals' names in the search in conjunction with terms such as homicide or suicide made missing data less likely.

It is possible that mental illness was underreported, either through the reporter's lack of interest in the offender's mental health history or because they were unable to access medical information. Websdale & Alvarez^25^ suggested that at the time of the incident the pivotal features of an article for journalists are ‘the crime-scene, the victims, and the aftermath of these killings’.

Homicide-suicide attracts a disproportionate amount of media attention. Although the number of incidents per year may be relatively small, the excessive and prominent newspaper reporting will inevitably influence our perception of these incidents and inform our understanding of the motivation for these acts. Previous research has shown an association between mental illness and homicide-suicide and this remains an important risk factor.^18^ Consequently, we would encourage the accurate reporting of mental illness in the media and advocating help-seeking behaviour in people who may be experiencing similar emotional distress. This is particularly important for men following the breakdown of a long-standing intimate relationship.^26^
